# The Draft Genome Sequence of European Pear (*Pyrus communis* L. ‘Bartlett’)

**DOI:** 10.1371/journal.pone.0092644

**Published:** 2014-04-03

**Authors:** David Chagné, Ross N. Crowhurst, Massimo Pindo, Amali Thrimawithana, Cecilia Deng, Hilary Ireland, Mark Fiers, Helge Dzierzon, Alessandro Cestaro, Paolo Fontana, Luca Bianco, Ashley Lu, Roy Storey, Mareike Knäbel, Munazza Saeed, Sara Montanari, Yoon Kyeong Kim, Daniela Nicolini, Simone Larger, Erika Stefani, Andrew C. Allan, Judith Bowen, Isaac Harvey, Jason Johnston, Mickael Malnoy, Michela Troggio, Laure Perchepied, Greg Sawyer, Claudia Wiedow, Kyungho Won, Roberto Viola, Roger P. Hellens, Lester Brewer, Vincent G. M. Bus, Robert J. Schaffer, Susan E. Gardiner, Riccardo Velasco

**Affiliations:** 1 Palmerston North Research Centre, The New Zealand Institute for Plant & Food Research Limited (Plant & Food Research), Palmerston North, New Zealand; 2 Mount Albert Research Centre, Plant & Food Research, Auckland, New Zealand; 3 Istituto Agrario San Michele all'Adige (IASMA) Research and Innovation Centre, Foundation Edmund Mach (FEM), San Michele all' Adige, Trento, Italy; 4 Lincoln Research Centre, Plant & Food Research, Lincoln, New Zealand; 5 School of Biological Sciences, University of Auckland, Auckland, New Zealand; 6 Institute of Food, Nutrition and Human Health, Massey University, Palmerston North, New Zealand; 7 Institut de Recherche en Horticulture et Semences (IRHS), Institut National en Recherche Agronomique (INRA), Angers, France; 8 National Institute of Horticultural and Herbal Science (NIHHS), Rural Development Administration (RDA), Naju, Republic of Korea; 9 Motueka Research Centre, Plant & Food Research, Motueka, New Zealand; 10 Hawke's Bay Research Centre, Plant & Food Research, Havelock North, New Zealand; Agriculture and Agri-Food Canada, Canada

## Abstract

We present a draft assembly of the genome of European pear (*Pyrus communis*) ‘Bartlett’. Our assembly was developed employing second generation sequencing technology (Roche 454), from single-end, 2 kb, and 7 kb insert paired-end reads using Newbler (version 2.7). It contains 142,083 scaffolds greater than 499 bases (maximum scaffold length of 1.2 Mb) and covers a total of 577.3 Mb, representing most of the expected 600 Mb *Pyrus* genome. A total of 829,823 putative single nucleotide polymorphisms (SNPs) were detected using re-sequencing of ‘Louise Bonne de Jersey’ and ‘Old Home’. A total of 2,279 genetically mapped SNP markers anchor 171 Mb of the assembled genome. *Ab initio* gene prediction combined with prediction based on homology searching detected 43,419 putative gene models. Of these, 1219 proteins (556 clusters) are unique to European pear compared to 12 other sequenced plant genomes. Analysis of the expansin gene family provided an example of the quality of the gene prediction and an insight into the relationships among one class of cell wall related genes that control fruit softening in both European pear and apple (*Malus*×*domestica*). The ‘Bartlett’ genome assembly v1.0 (http://www.rosaceae.org/species/pyrus/pyrus_communis/genome_v1.0) is an invaluable tool for identifying the genetic control of key horticultural traits in pear and will enable the wide application of marker-assisted and genomic selection that will enhance the speed and efficiency of pear cultivar development.

## Introduction

Pear (genus *Pyrus*) is one of the oldest temperate tree fruit crops, having been grown since antiquity from both Europe to China. Homer described the pear in the ‘Odyssey’ as a “gift of the gods”. Pear production was approximately 23.9 MT worldwide in 2012 (http://faostat3.fao.org/), with European pear (*Pyrus communis* L.; 2*n* = 34) making up about one third of total production. The genus *Pyrus* is related to apple (*Malus*) and quince (*Cydonia*) within the tribe *Pyreae* [1], which all share the pome fruit structure. Pear has historically been less well researched than other members of the Rosaceae such as apple, peach and strawberry. Recently, whole-genome sequences have been developed for a range of economically important dicotyledonous plants, such as poplar, grape, papaya, cucumber, cocoa, potato, soybean, cannabis, melon and tomato [2]–[15], including the rosaceous crops apple, strawberry, peach and Chinese pear (*P. bretschneideri*) [16]–[19]. Low to medium density pear genetic maps enriched with apple microsatellite markers have enabled the alignment of genetic maps of European pear and apple and formulation of the hypothesis that apple and pear have collinear genomes [20]–[24]. Although this hypothesis was based on few hundred orthologous markers only, the recent comparison of several sequenced rosaceous genomes indicates that even among the more distantly related genomes of apple, peach and strawberry [25], [26], synteny is conserved. It might be anticipated that the synteny between apple and pear should be higher than in these cases, as apple and pear are more closely related phylogenetically than apple is to peach and strawberry [1]. We have taken advantage of the current cost and effectiveness of genome sequencing technologies to develop the genome assembly of European pear, with the ultimate goal of developing an understanding of the traits that differentiate the more distantly related rosaceous crops, as well as those more closely related within the *Pyreae*. European pear has several biological features that differentiate it from apple and Chinese pear, such as traits controlling melting fruit flesh versus crisp flesh, and species-specific susceptibility to pests and pathogens. We wish to compare the European pear genome with that of apple and Chinese pear, for the purpose of developing ultimately an understanding of the evolution of the core traits that differentiate apple and pear, as well as the control of the very different flesh types and flavours between European and Chinese pears.

We chose ‘Bartlett’ (also known as ‘William's Bon Chrétien’ or ‘William's pear’) for genome sequencing, not only because of its major role as a cultivar in Europe, but also because it is a founder of most *P. communis* breeding programmes worldwide. The draft genome assembly of European pear was developed using Roche 454 sequencing technology and spans 577.3 Mb, containing 43,419 putative genes. We tested the integrity of the assembly by examining the expansin gene family, members of which are involved in fruit ripening of pome fruit, as an example of the type of insights into functional biology that can be achieved using this genome sequence.

## Methods

### Plant material and nucleic acid extraction

DNA was extracted from young leaves of *P. communis* ‘Bartlett’ grown at the Plant & Food Research (PFR) Motueka research orchard (New Zealand; 41°8′0″ South, 173°1′0″ East) and in Field 11.C of Maso Parti at Edmund Mach Foundation-Istituto Agrario di San Michele all'Adige (Italy; 46°12′ North, 11°8′ East) (no permission was required to collect these samples and they are not from endangered or protected species), using the QIAGEN DNeasy Plant Kit (QIAGEN GmbH, Hilden, Germany). DNA quality was assessed by agarose gel electrophoresis to ensure that DNA was not degraded. Expression analysis was undertaken on *P. communis* ‘Doyenne du Comice’ (‘Comice’) and *P. pyrifolia* ‘Nijisseiki’ pears grown at PFR, Motueka (New Zealand) harvested at standard commercial ripeness (‘Comice’: firmness <5.5 Kg.F, and partial starch clearance; ‘Nijisseiki’: total starch hydrolysis) and stored for 8 weeks at 0.5°C. Following cold storage, fruit were left at 20°C for 7 days, to allow the fruit to soften, before harvest into liquid N_2_ and storage prior to RNA extraction as described in [27] and cleaned with RNeasy cleanup columns (QIAGEN) following the manufacturer's instructions.

### Libraries and 454 pyrosequencing

Two random shotgun ‘genomic’ libraries were generated via fragmentation of 500 ng each of pear genomic DNA employing the GS FLX+ *Series* XL+ Rapid Library preparation kit, following the manufacturer's recommendations (Roche, Indianapolis, IN, USA). Three 2 kb and two 7 kb paired-end libraries were constructed from pear genomic DNA using the GS FLX+ *Series* XLR70 Paired End Rapid Library preparation kit following the manufacturer's recommendations (Roche). Five and 15 µg of double-stranded genomic DNA was randomly fragmented via hydrodynamic shearing to an average size of 2,000 and 7,000 bp using the HydroShear apparatus (DigiLab, Marlborough, MA, USA). The libraries were quantified by quantitative PCR using the 454 Kapa Library Quantification Kit (Kapa Biosystems, Boston, MA, USA). Long sequencing reads from shotgun ‘genomic’ libraries and paired-end sequencing reads were produced by the GS FLX+ *Series*, using the GS FLX Titanium Sequencing Kit XL+ (Roche), according to the manufacturer's recommendations.

### mRNA-seq analysis

For each sample, ten micrograms of RNA was sequenced to a depth of ∼20M reads using Illumina Hi-Seq contracted through Macrogen (Seoul, Korea; www.macrogen.com). Frequency counts were obtained using *Bowtie2* [28] to align reads to the predicted gene models detailed below. Reads Per Kilobase per Million (RPKM) mapped reads were extracted from the BAM files using the ‘DEseq’ library in Bioconductor (www.bioconductor.org) in the statistical software package ‘R’. Quantitative PCR (qPCR) was performed as described in [29], with *Actin* as a control, using primers MdEXPA2F (TTCCAAGACAGGGTGGCAAG) and MdEXPA2R (TGCCCTCAAATGTTTGTCCG) for apple and PcEXP2F (GGCAAGCCCTGTCAAGAAAT) and PcEXP2R (GCCCTCAAATGTTTGTCCG) for pear.

### Genome assembly

GS FLX+ reads were assembled with the Roche GS De Novo Assembler (version 2.7; http://454.com/products/analysis-software/index.asp), using both the large and heterozygous genome modes and 8 CPUs. All other assembler configuration settings were left at their default settings. The completeness of the assembly was estimated by Core Eukaryotic Genes Mapping Approach (CEGMA) analysis (version 2.4.010312) [30].

### Genome anchoring

Four segregating populations of pear were genotyped using the apple and pear single nucleotide polymorphism (SNP) array [31], [32]. The families consisted of one *P. communis* intra-specific population and three inter-specific Asian×European pear populations: ‘Old Home’×‘Louise de Bonne Jersey’ (297 F1 individuals), NZSelection_pearT003×‘Moonglow’ (92 F1 individuals), NZSelection_pearT042×NZSelection_pearT081 (142 F1 individuals) and NZSelection_pearT052×NZSelection_pearT003 (91 F1 individuals) [32]. The Asian parents (of complex Chinese and Japanese pear origin involving both *P. bretschneideri* and *P. pyrifolia*) and inter-specific hybrid populations were developed and maintained at PFR, Motueka. Three segregating populations of apple (PremA153×NZSelection_appleT031, ‘Fuji’×NZSelection_appleT051 and ‘Sciros’×NZSelection_appleT051) [33] were used to construct the apple genetic maps. These were developed for each parent of the respective populations using Joinmap v3.0 (www.kyazma.nl). Markers were anchored to the ‘Bartlett’ genome assembly v1.0 (Bartlett v1.0) using BLAST-like alignment tool (BLAT) analysis [34] by searching for scaffolds with similarity to the flanking sequence of the pear and apple SNPs. Figure S1 outlines the strategy employed for genome anchoring.

### Gene prediction and annotation

*De novo* assembly of ‘Comice’ transcripts was performed using trans-ABySS (v1.3.2) [35]. Briefly, 58,026,953 Illumina HiSeq RNASeq reads were trimmed by 15 bases at their 5′ ends, filtered to remove reads containing ambiguities using an in-house PERL script. The RNASeq reads were subsequently trimmed to a minimum quality score of 20 using the program fastq-mcf from the ea-utils package (http://code.google.com/p/ea-utils). Transcript contigs resulting from *de novo* assembly using every second kmer from 35 to 69 were then merged in to a single transcript set with the program abyss-rmdups-iterative from the trans-ABySS software distribution.

Gene prediction used a hybrid prediction approach, combining *ab initio* gene prediction and homology searching. Specifically Augustus (Augustus 2.7) trained using the ‘Comice’ transcripts was employed for gene prediction *ab initio* from European pear scaffolds. Augustus predictions were performed separately on unmasked and repeat masked scaffolds. RepeatMasker (version 4-0-3 [36]) was employed to mask known repeats in the genome scaffolds using the rosid clade of repeats from RepBase (Update 20120418, RM database version 20120418) and rmblastn version 2.2.27+ (ftp://ftp.ncbi.nlm.nih.gov/blast/executables/rmblast/2.2.27/). Homology searching was performed by comparison with predicted proteins from other Rosaceae. Predicted proteins were obtained for apple (http://genomics.research.iasma.it/), Chinese pear (http://peargenome.njau.edu.cn:8004/), peach (http://www.rosaceae.org/sites/default/files/peach_genome/Prunus_persica_v1.0_peptide.fa.gz) and strawberry (http://www.rosaceae.org/sites/www.rosaceae.org/files/strawberry/genome/v1.0/fvesca_v1.0_genemark_hybrid.faa.gz). These rosid protein sequences were compared to repeat-masked European Pear scaffolds using TBLASTN [37]. Alignment results were filtered using a modified version of blast92gff3.pl (http://iubio.bio.indiana.edu/gmod/tandy/perls/blast92gff3.pl), to identity sequences with greater than 79% identity and to mediate running GeneWise (wise-2.4.1; [38]) on the retrieved region, as well as 1000 bases upstream and downstream of the aligned regions. GeneWise predictions were assessed using evigene (http://marmot.bio.indiana.edu/EvidentialGene/) and the best models (evigene's ‘okayset’) retained. Where a model from more than one approach was present at any locus, the model representing the cluster was selected on the basis of homology to proteins from Swissprot and rosid species, as well as prediction length. Models from predictions on the unmasked gene for which there was no supporting model from the GeneWise or masked genome predictions were excluded from the final gene model set. However, models from masked, unmasked and hybrid approach predictions were separately annotated using Plant & Food Research's in-house BioView Sequence Analysis and Annotation pipeline [39] and results for each prediction set have been made available as a track in the genome browser (http://www.rosaceae.org/species/pyrus/pyrus_communis/genome_v1.0). BioView annotated the predicted gene models by searching the Swissprot, Uniref90 (http://www.uniprot.org/downloads) [40], RefSeq (release 54) [41], and *Arabidopsis* proteins (TAIR 10) databases using BLASTX (version 2.2.25) [37]. Searching against the NCBI non-redundant (NR) DNA database (ftp://ftp.ncbi.nlm.nih.gov/blast/db/) was performed using BLASTN (version 2.2.25) [37], while Gene Ontology terms were derived following motif searching based on InterproScan (version 4.8) [42] and Interpro Release 38 (http://www.ebi.ac.uk/interpro/). Comparison of metrics for European pear gene models to that for apple, Chinese pear and strawberry was performed as follows. Published GFF3 files describing gene models for apple and strawberry were obtained from the Genome Database for Rosaceae (GDR) (http://www.rosaceae.org/) and those for Chinese pear from http://peargenome.njau.edu.cn:8004. An in-house PERL script was used to parse the GFF3 files and extract metrics from each set. The extracted metrics will be influenced by the different gene model prediction methodologies used by the different authors and should be considered with this caveat in mind.

### Comparative analysis of proteomes

The predicted European pear protein sequences were compared with those from apple v1.0 (http://genomics.research.iasma.it/), Chinese pear v1.0 (http://peargenome.njau.edu.cn:8004/), strawberry v1.1 (http://www.rosaceae.org/species/fragaria/fragaria_vesca/genome_v1.1), grape v1.0 (http://genomics.research.iasma.it/), kiwifruit (http://bioinfo.bti.cornell.edu/cgi-bin/kiwi/download.cgi), poplar v3.0 (ftp://ftp.jgi-psf.org/pub/JGI_data/phytozome/v8.0/early_release/Ptrichocarpa_v3.0/), sweet orange v1.0 (http://www.citrusgenomedb.org/), mandarin v1.0 (http://www.citrusgenomedb.org/), papaya v1.0 (ftp://asgpb.hawaii.edu/papaya/), tomato v1.0 (ftp://ftp.sgn.cornell.edu/genomes/Solanum_lycopersicum/assembly/current_build/), potato v4.03 (http://solanaceae.plantbiology.msu.edu/pgsc_download.shtml), and *Arabidopsis* (TAIR 10; http://www.arabidopsis.org/), to identify ortholog gene clusters. These published datasets were developed using different genome annotation strategies, utilizing different tools. Although, each plant genome may hence contain biases of various types, we consider these data acceptable for application in our comparative study.

Protein sequences shorter than 10 amino acids and those containing more than 20% stop codons were excluded from the analysis. The remaining sequences were reciprocally blasted against each other using BLASTP with cut-off e value 1e-10. The similarity calculation, in-paralog and co-ortholog analyses were performed using Orthomcl-2.0.3 [43] together with mcl-09-149 (http://micans.org/mcl/). A visualized summary of ortholog clusters between 13 plant species was generated with in-house PERL and R scripts.

### Estimating phylogenetic relationships

Phylogenetic trees were constructed based on protein sequences of 83 “euKaryote Orthologous Genes” (KOGs). Multiple sequence alignments were performed using MUSCLE v3.8.31. Well-aligned regions were extracted with GBLOCKS 0.91b. The maximum-likelihood phylogenetic calculation was performed using PhyML with the Blosum62 amino acid substitution model and 100 rapid bootstrap partitions. The tree was visualized using Figtree 1.4.0.

### Expansin gene family analysis

The expansin gene family was chosen for further analysis, to support the completeness of the gene predictions for European pear, as well as to examine the degree of similarity in the gene space between the apple and European pear genomes. Expansin protein sequences from apple and *Arabidopsis* were used to perform a BLASTP search against the apple predicted peptide models, in order to identify putative expansins with a BLAST score >50. The corresponding expansin-like genes from apple were then used in a BLASTP search against the pear peptide models. Protein sequences were aligned in Geneious 6.1.6 (Biomatters Ltd, Auckland, NZ) using Geneious alignment with Blosum45 cost matrix. From this alignment, genes were further filtered by selecting those containing conserved expansin domains as classified by [48] with a conserved region of similarity corresponding to 313 residues and used to create a phylogenetic tree derived using the maximum likelihood Geneious plug-in, PhyML with the JTT substitution model and bootstrap analysis of 1000 data sets. *DdEXP2* from the amoeba *Dictyostelium discoideum* was used as an outgroup [44].

### *De Novo* repeat annotation

The genomic scaffolds of the ‘Bartlett’ v1.0 and the primary assembly of ‘Golden Delicious’ were analysed using RepeatScout [45] to provide *de novo* a list of repetitive elements independent of repeats identified by repeat masking using RepeatMasker and RepBase. The list was further analysed for redundancy and classified into repeat classes using TEclass [46].

### SNP detection

The pipeline used for SNP discovery in European pear was similar to that described for apple [31]. Genomic DNA was extracted from *P. communis* cultivars ‘Louise Bonne de Jersey’ (LBJ) and ‘Old Home’ (OH) grown at PFR, Motueka (no permission was required to collect these samples and they are not from endangered or protected species) using the QIAGEN DNeasy Plant Kit (QIAGEN) and sequenced using one lane of Illumina® GA II with 75 cycles per read [32]. Reads were aligned to Bartlett v1.0 scaffolds using Soap2.2.1 [47]. SNPs were detected using SoapSNP (http://soap.genomics.org.cn/soapsnp.html) essentially as described in [48]. Genome partitioning of SNPs was based on the location of predicted gene models.

## Results

### Genome sequencing and assembly of Bartlett v1.0

In total, 23,058,965 paired-end (43.7%) and non paired-end (56.3%) sequence reads yielded 8.2 Gigabases (Gb) of sequences (Table S1) that were used to develop the *P. communis* ‘Bartlett’ genome assembly v1.0 (Bartlett v1.0) (Table 1). The estimated genome size based on flow cytometry [49] is approximately 600 Mb of haploid genome, and our data enable estimation of a 11.4× average coverage. The assembly gave 182,196 contigs of a cumulative length of 507.6 Mb. These contigs were assembled into scaffolds using a combination of Roche 454 2 kb and 7 kb insert library paired-end reads to obtain 142,083 Bartlett v1.0 scaffolds, covering a total of 577.3 Mb, and representing most of the haploid *P. communis* genome. The longest scaffold was 1.2 Mb long and 50% of the assembled genome was contained in 1,442 scaffolds (L50), with the smallest L50 scaffold comprising 88,114 bp (N50). Only 12.1% of the scaffold sequences were unknown bases. The completeness of the draft genome assembly was tested by searching for 248 Core Eukaryotic Genes (CEGs; [30]). In total, 232 of 248 (93.5%) CEGs were completely present and 244 of 248 CEGs were completely or partially present (98.4%) (Table S2).

**Table 1:** Basic statistics on the *Pyrus communis* ‘Bartlett’ genome sequence.

**Sequencing data**	
Number of bases used for assembly	8,204,442,728
Sequenced reads used for assembly	23,058,965
Non paired end	12,979,485
Paired end	10,079,480
Estimated average coverage*	11.4×
**Contigs**	
Number of contigs	182,196
Total size of contigs (bp)	507,689,959
N50 contig length (bp)	6,569
Longest contig (bp)	127,414
Mean contig size (bp)	2,787
Median contig size (bp)	1,188
Number of contigs in scaffolds	47,404
Number of contigs not in scaffolds	134,792
**Scaffolds**	
Number of scaffolds	142,083
Total size of scaffolds (bp)	577,335,413
N50 scaffold length (bp)	88,114
Longest scaffold (bp)	1,291,680
Shortest scaffold (bp)	501
Number of scaffolds >1K bp	69,460
Number of scaffolds >10K bp	4,916
Number of scaffolds >100K bp	1,262
Number of scaffolds >1M bp	4
Number of scaffolds >10M bp	0
Mean scaffold size (bp)	4,063
Median scaffold size (bp)	983
Scaffold %N	12.06%

Figures are given in bp.

*: the assumed genome size of pear is 600 Mb.

### Genome anchoring to pear and apple genetic maps

The scaffolds of Bartlett v1.0 were anchored to high density genetic maps constructed for *Pyrus* [32] and *Malus* segregating populations [33] using SNP markers from the International RosBREED SNP Consortium (IRSC) apple and pear array [31], [32]. The IRSC array contains 7,692 *Malus* SNPs, as well as 1,096 SNPs developed from *P. communis*. In total, 2,279 genetically mapped loci (1,391 and 888 apple and pear SNPs, respectively) yielded a significant BLAT hit to 868 unique scaffolds (Table 2), enabling the anchoring of a total of 171.3 Mb of the assembled genome to the 17 *Pyreae* LGs (Table S3). The largest LG was LG15 (17.6 Mb) and the median number of markers per scaffold was 2.0.

**Table 2:** Anchoring of the *Pyrus communis* ‘Bartlett’ assembly v1.0 genome sequence.

LG	Length anchored (bp)	Number of anchored scaffolds (unique)	Number of anchoring markers	Median number of markers per scaffold
1	8,550,412	46	115	2.0
2	11,234,491	58	194	3.0
3	12,642,036	69	163	2.0
4	8,044,179	40	105	2.0
5	10,949,710	57	159	2.0
6	8,104,341	45	117	1.0
7	8,833,777	53	102	1.0
8	8,189,737	36	92	2.0
9	10,984,512	53	145	2.0
10	9,331,439	54	113	2.0
11	10,224,161	53	134	2.0
12	8,857,939	44	122	2.0
13	10,282,711	38	127	2.5
14	10,094,382	51	117	2.0
15	17,650,274	75	222	2.0
16	8,177,493	44	124	2.0
17	9,204,799	52	128	2.0
Total	171,356,393	868	2,279	2.0

### Gene prediction

Gene prediction using a combined *ab initio* prediction and homology searching approach yielded 43,419 putative gene models (Table 3). The number of predicted genes is higher than for most plant species and ∼30% greater than in the strawberry genome (34,809 gene models), as might be expected due to the *Pyreae* whole genome duplication [17]. The average predicted coding region length (1,209 bp) was similar to that in Chinese pear, strawberry and apple (Table 3), as was the average predicted exon length between the predicted protein sets from these four rosaceous species. These similarities are observed in spite of the different gene model prediction methodologies utilized, and which should be taken into account when considering these observations. The number of single exon genes was similar between European and Chinese pears as well as apple, at about twice that of strawberry. The gene density in European pear was estimated to be 7.5 genes per 100 kb which is similar to that for Chinese pear, apple (Table 3), poplar (9.4 [10]), grape (6.6 [12]) and melon (7.3 [3]), but not as dense as observed for strawberry (14.5 [16]), notwithstanding the methodological difference in gene prediction employed for each species.

**Table 3:** Gene prediction summary for *Pyrus communis* and comparison with *P. bretschneideri*, *Fragaria vesca* and *Malus*×*domestica*.

	*Pyrus communis*	*Pyrus bretschneideri*	*Fragaria vesca*	*Malus*×*domestica*
Predicted genes	43,419	42,812	34,809	54,921
Average gene length (including introns)(nt)	3,320	2,776	2,792	2,802
Average CDS length (nt)	1,209	1,172	1,160	1,155
Exons	221,804	202,169	174,376	273,226
Average exon length	237	248	232	273
Single exon genes	10,909	12,310	5,915	10,378
Introns	178,385	159,357	139,567	218,353
Introns per gene (multi-exon genes only)	5.49	5.22	4.83	4.90
Average intron length	398	386	409	491
Genes per 100 Kb	7.5	8.4	14.5	7.3

Gene predictions were performed using Augustus for European pear and GeneMark-ES for strawberry. The apple gene models were estimated as the total number of gene predictions minus an estimation of duplications generated by contig overlaps. The redundancy was filtered out using similarity among predictions and positional considerations.

**Figure 1:**
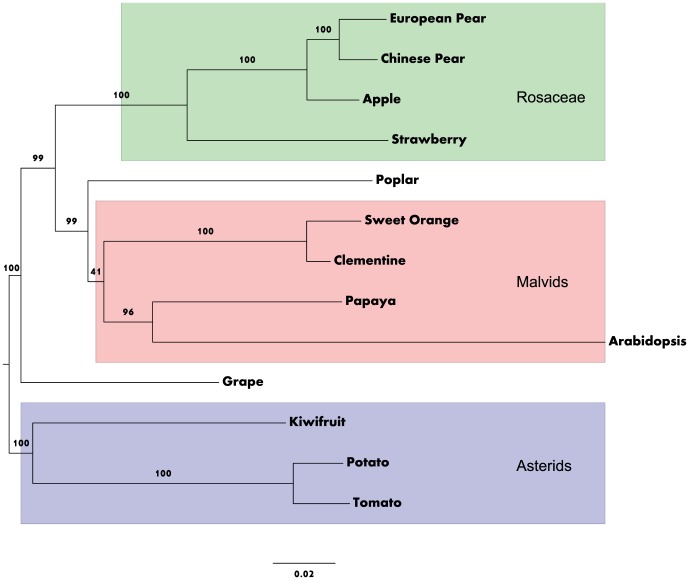
Phylogenetic tree of six rosids, four malvids, and three asterids constructed with 83 euKaryote Orthologous Genes (KOGs). Bootstrap values are listed on each branch. Nodes represent speciation events and branch length represents the degree of evolutional changes over time. The unit for the scale bar at the bottom is nucleotide substitutions per site. The high bootstrap values strongly support that the species in Rosaceae cluster together to the exclusion of any other, and that the European pear and Chinese pear separation event happened after apple speciation.

A phylogenetic tree constructed with 83 euKaryote Orthologous Genes (KOGs) in six rosids, four malvids, and three asteroids (Figure 1) confirmed that European pear is a close relative of Chinese pear and apple and is more distantly related to strawberry.

### Comparative analysis of proteomes

**Figure 2:**
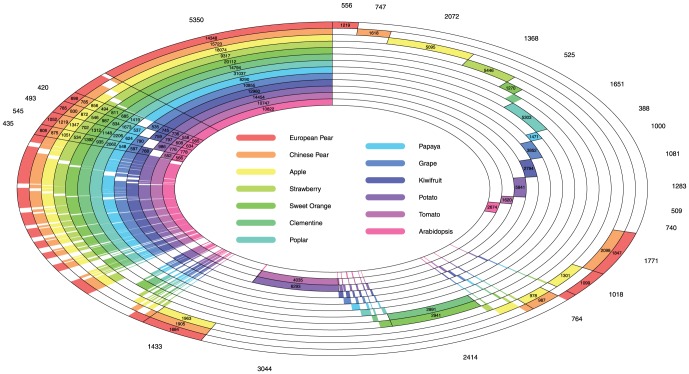
Protein-protein comparison between European pear and 12 other species: Chinese pear, apple, grape, strawberry, papaya, sweet orange, clementine, kiwifruit, tomato, potato, poplar and *Arabidopsis*. The figure shows every possible combination of species included in this proteome ortholog analysis, using concentric circles. Each ring represents a single plant species and is depicted in a unique colour. For the 13 species shown, there are hence a total of 2^13^–1 combination cases, from 556 ortholog groups found in European pear only, 682 clades in Chinese pear only, to 5393 clusters present in all thirteen species. For each combination, the number of ortholog groups discovered is labelled outside the outermost ring and the number of proteins for a species inside a coloured, circular cell that represents the particular species. As the angular width of the cells for each case is drawn proportional to its number of groups, there is no labelling where the angular width is too small. A complete list of all combination cases with detected ortholog genes is provided in Table S4.

A total of 5,350 protein clusters was observed as conserved across all 13 species proteomes, with 14,348 predicted European pear proteins (33% of the 43,419 total predicted protein set; Figure 2). Only 82 protein clusters were not found in European pear compared with all other 12 species, a value less than the number of protein clusters absent from Chinese pear (298), apple (236), strawberry (192), *Arabidopsis* (246), potato (437), papaya (424), grape (502) and kiwifruit (558), however similar to that of sweet orange (85), clementine (34), tomato (53) and poplar (45) (Table S4). The proteome analysis demonstrates close genome relatedness between Chinese pear, European pear and apple; tomato and potato; sweet orange and Clementine, respectively. More protein clusters were shared between European and Chinese pear (1,771), than those between Chinese pear and apple (764) and between European pear and apple (1,018). There are 1,433 groups of orthologous protein clusters present in all the three species of the *Pyreae*. These share the highest number of unique ortholog groups in our analysis (5,552 in total), followed by *Solanaceae* with 3,044 clusters of 6,293 genes in potato and 4,035 genes in tomato, respectively, and by citrus (2,941 sweet orange genes and 2,991 clementine genes in 2,414 clusters). Finally, 556 clusters were unique to European pear and these corresponded to 1,219 proteins (2.8% of the 43,419 total predicted protein set; Table S5).

### Repeat analysis

A total of 199.4 Mb of repeated elements was identified in the unmasked Bartlett v1.0 genome scaffolds employing *de novo* detection followed by a classification made using RepeatMasker (Table 4). The most common repeated elements were long terminal repeat (LTR)/*Gypsy* (84.6 Mb; 14.1% of the assembled genome) and LTR/*Copia* (42.8 Mb; 7.1% of the assembled genome), and the most common DNA transposable elements (TEs) were PIF-*Harbinger* (10.2 Mb; 1.7% of the assembled genome) and *hAT-Ac* (4.7 Mb; 0.8% of the assembled genome). These results are in agreement with the analysis of the *P. bretschneideri* genome [18]. The classification of repeated elements using an homology-based search using the Rosaceae clade from RepBase (Table 5) confirms the results obtained by *de novo* detection, as LTR/*Gypsy* and LTR/*Copia* were the most abundant classes of retroelements. In total, 194.8 Mb (32.5%) of the assembled Bartlett v1.0 genome comprised interspersed repeated elements according to the homology-based analysis.

**Table 4:** Comparison of *de novo* predicted repeated elements between the European pear, Chinese pear and apple genomes. na: not available from [18].

	European Pear		Chinese pear	Apple	
Class	Count	Size(bp)	Size (bp)	Count	Size(bp)
DNA	87,258	16,034,061	19,622,007	62,032	12,036,031
DNA/CMC-EnSpm	5,245	2,498,065	1,994,298	3,238	1,651,963
DNA/MULE-MuDR	11,081	1,654,134	2,812,256	8,411	1,299,397
DNA/PIF-Harbinger	33,920	10,195,913	13,681,892	22,675	6,976,753
DNA/hAT-Ac	18,863	4,763,497	10,510,482	15,194	5,286,418
DNA/hAT-Tag1	8,271	1,973,418	2,689,092	10,877	3,050,698
DNA/hAT-Tip1	9,603	2,394,869	3,298,090	7,398	2,034,152
LINE/L1	8,410	4,077,094	10,075,654	5,716	3,269,671
LINE/RTE-BovB	7,697	2,200,632	4,408,634	12,750	10,996,014
SINE	425	124,106	208,975	2,191	359,108
SINE/tRNA	20,796	2,863,949	na	13,153	1,805,785
LTR	37,405	9,249,657	38,166,045	20,479	6,282,994
LTR/Caulimovirus	2,796	2,550,963	2,625,049	1,227	1,544,695
LTR/Copia	73,727	42,805,711	86,429,855	46,798	35,591,207
LTR/Copia-like	110	20,863	na	97	26,513
LTR/Gypsy	145,766	84,633,023	130,449,009	94,218	68,540,726
Low_complexity	121,061	5,222,319	na	84,700	3,780,221
RC/Helitron	6,851	2,104,677	na	5,036	1,448,948
Satellite	207	30,594	350,647	178	40,455
Simple_repeat	63,595	2,428,632	1,131,844	47,558	1,891,325
Unknown	6,610	1,545,468	4,296,548	4,650	1,079,420
rRNA	351	100,288	na	158	31,199
**TOTAL**		**199,471,933**	**332,750,377**		**169,023,693**

**Table 5:** Classification of repeated elements in European pear based on the ‘Rosaceae’ clade from RepBase.

	Copies	Assembled (Mb)	Assembly %
**Retroelements**	241,316	149.345	25.87
SINEs	20,442	2.865	0.50
LINE/RTE	6,669	1.724	0.30
LINE/L1	7,851	4.300	0.74
LTR/Ty1/Copia	56,724	44.240	7.66
LTR/Gypsy	114,127	85.382	14.79
**DNA transposons**	173,387	42.058	7.28
hobo-Activator	36,417	10.007	1.73
Tc1-IS630-Pogo	107	0.015	0
En-Spm	296	0.278	0
Tourist/Harbinger	30,859	9.770	1.69
**Unclassified**	11,995	3.427	0.59
**Total interspersed repeats**		194.830	33.75
**Small RNA**	20,415	2.873	0.50
**Satellites**	186	2.873	0.01
**Simple repeats**	2,497	0.021	0.04
**Low complexity**	226	0.000	0.00
**TOTAL**		197.724	34.14

### SNP detection

Sequencing of LBJ and OH yielded 25,167,853 and 35,687,533 paired end reads, representing approximately 6.6× and 9.2× coverage per genotype, respectively. A total of 3,893,643 putative SNPs was identified following mapping of LBJ and OH low coverage sequencing data to the Bartlett v1.0 assembly scaffolds. Of these 829,823 (21.3%) passed the filtering condition for stage 1 detection defined in [31]. The average SNP frequency of SNPs passing the filtering conditions was one per 674 bp with 146,585 (17.7%) predicted to be located within exons in the predicted gene models. A further 60,820 (7.53%) and 51,425 (6.37%) SNPs were located within 1,000 bases upstream or downstream of a predicted gene model, respectively.

### Insight into the European pear annotated genome: example of the expansin gene family

**Figure 3:**
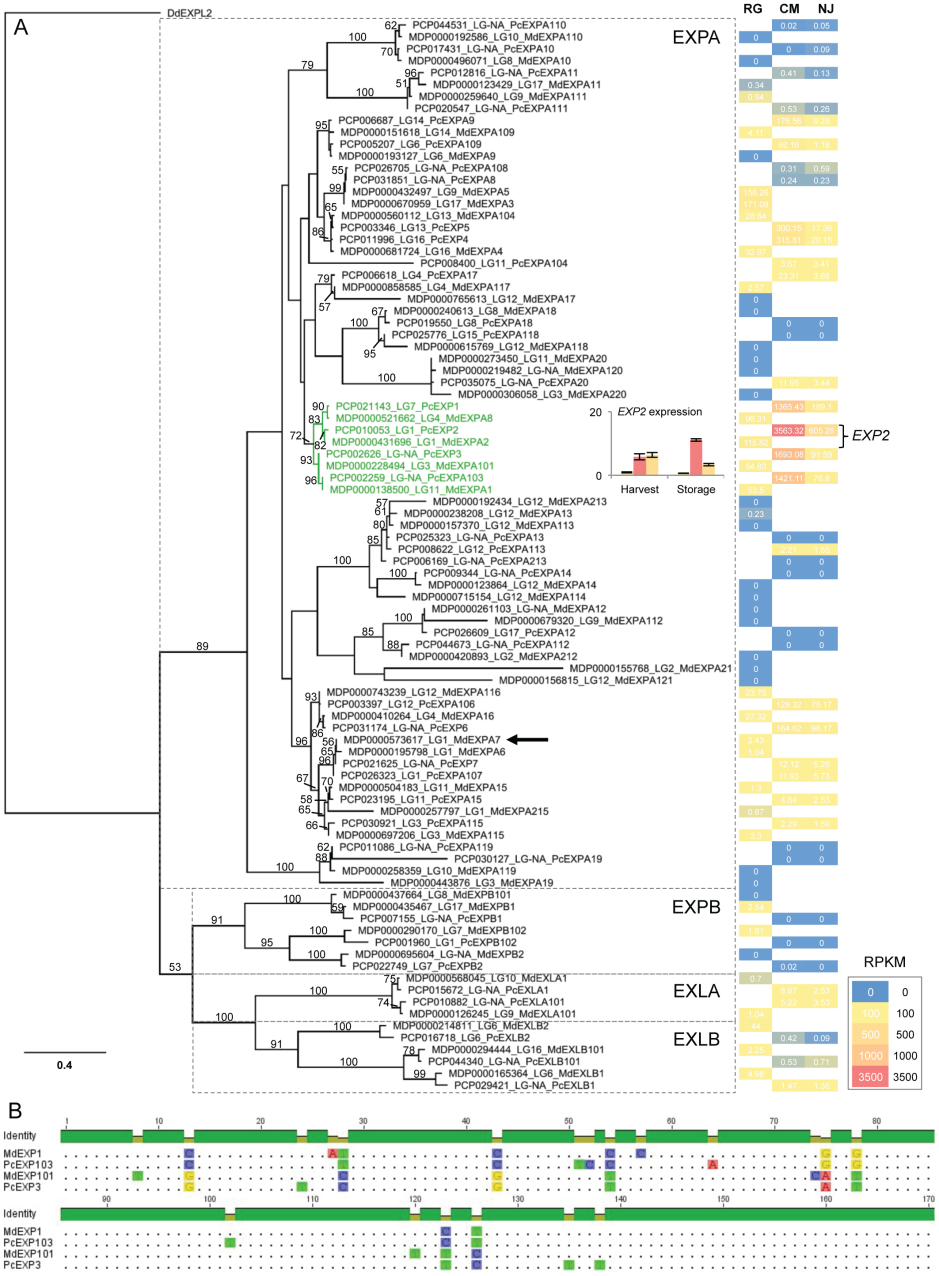
Phylogenetic and gene expression analysis of the expansin-like genes from apple and European pear. A) Phylogenetic tree of predicted expansin-like genes from apple and European pear. Predicted expansin-like protein models from apple (MDP prefix) and European pear (PCP prefix) were aligned, and a conserved region of alignment of 313 residues was used to construct the phylogenetic tree Geneious 6.1.6 (Biomatters Ltd, Auckland, NZ). The linkage group (LG) of each model is shown where possible; some models are not anchored (LG-NA) to the genome. Models that represent the best hit for published expansins are labelled additionally as such. *DdEXP2* from *Dictyostelium discoideum* was used as an out-group. Bootstrap proportions for 100 trees were calculated and bootstrap values ≥50 are shown. Scale indicates 0.4 substitutions per site. EXPA, α-expansins; EXPB, β-expansins; EXLA, alpha-like expansins; EXLB, beta-like expansins [50]. mRNA-seq expression levels in ‘Comice’ melting pear (CM), ‘Nijisseki’ (NJ) crisp pear and ‘Royal Gala’ (RG) crisp apple, undergoing fruit ripening in storage show that one clade is strongly associated with fruit ripening (coloured green). The inserted graph shows the expression analysis by qPCR of *EXP2* in fruit at harvest and during storage, which corresponds to the mRNA-seq data. Yellow bars: RG, red bars CM, orange bars NJ). RPKM: Reads Per Kilobase per Million mapped reads. Single arrow shows the apple expansin (MdEXPA7) mapped to a quantitative trait locus for fruit texture. B) Alignment of the first 170 bp of apple and pear homologues, demonstrating genome duplication preceded speciation.

In total, 49 and 41 apple and pear expansin-like genes were identified respectively in predicted gene sets, and were accepted or rejected for inclusion in the phylogenetic analysis based on previously published expansin classification criteria [48] (Figure 3). Nine apple gene models did not have orthologous gene models in European pear and one additional pear gene model was identified with no apple ortholog (PCP008400). The predicted expansin and expansin-like genes from pear and apple grouped into four major clades, corresponding to the α- and β-expansins (EXPA and EXPB, respectively) and the two expansin-like families, EXPANSIN-LIKE A (EXLA) and EXPANSIN-LIKE B (EXLB) [50] (Figure 3A; Table S6). Homeologous genes derived from the *Pyreae* whole genome duplication were identified for both apple and European pear. Expansin genes within sub-clades showed more similarity between apple and pear orthologs, than between homeologues of the same species, confirming that speciation happened after the genome duplication event (Figure 3B).

For the rapidly softening European pear ‘Comice’ and crisp textured ‘Nijisseki’ (Japanese pear) 18.8M and 19.7M mRNA reads were obtained, respectively. Expression levels of the expansin class of genes determined in cold-stored ‘Comice’ and ‘Nijisseiki’ pears that were undergoing rapid softening were aligned to the phylogenetic clusters. These were compared to previously published mRNA-seq data mapped to the apple gene models [17] from mature, ripening ‘Royal Gala’ apples [51] (Figure 3A). It was observed that in most cases orthologous genes were expressed in both apple and pear during fruit ripening; however, the melting texture European ‘Comice’ pears exhibited a considerably higher level of expression than the crisp textured apples and ‘Nijisseiki’ Japanese pears, with some genes (such as *EXP2*) showing over 20-fold higher expression in ‘Comice’ compared with apple and ‘Nijisseiki’. qPCR of *EXP2* verified the mRNA-seq data and showed that at harvest and during storage, ‘Royal Gala’ exhibited consistently lower levels of *EXP2* expression than the pear varieties (Figure 3A).

## Discussion

### The draft genome assembly of Pyrus communis and its applications

We have used Roche 454 shotgun sequencing to develop the first draft genome assembly of European pear. European pear (*P. communis*) is the newest addition to the palette of whole genome sequences of Rosaceae fruit species, following apple (*Malus*×*domestica*; [17]), strawberry (*Fragaria vesca*; [16]), peach (*Prunus persica*; [19]) and Chinese pear (*P. bretschneideri* [18]). The Bartlett v1.0 draft genome spans most of the *P. communis* genome and 171 Mb is anchored to high density genetic maps. A total of 829,823 SNPs passed filtering criteria, which corresponds to one SNP every 674 bp. This SNP frequency in *P. communis* is lower than in apple (one SNP every 249 bp [46]), however, this may reflect the smaller set of cultivars used for SNP detection in European pear compared with apple. The development of a whole-genome sequence is a key milestone for research in any organism and the Bartlett v1.0 draft genome assembly will provide a springboard to explore the genetic control of key horticultural characters such as fruit quality, pest and disease resistance, and tree architecture. The genome assembly also enables the development of genetic markers for early selection of seedlings carrying alleles conferring these traits, from breeding germplasm. This genomic resource is now available to fruit researchers at the Genome Database for Rosaceae (http://www.rosaceae.org/species/pyrus/pyrus_communis/genome_v1.0). The number of predicted gene models (43,419), the high proportion of CEG retrieved (98.4%), and the comparison of apple and pear gene models of the expansin-like gene family demonstrate the quality and the completeness of the Bartlett v1.0 draft genome. A further valuable objective of developing a genome, beyond mining genes for sequence variants for linkage analysis, is to identify gene features such as open reading frames, introns and promoters for functional analysis. Although the Bartlett v1.0 draft genome sequence is fragmented, we have shown that it is sufficiently complete to enable functional characterisation of pear genes. Furthermore, our analysis of the Bartlett v1.0 draft genome indicated that European and Chinese pear have similar genome composition in terms of repeated elements, for example the LTR *gypsy* and *copia* elements are the most highly represented classes in both species. One striking feature of the pear genome is that it is smaller than that of apple, based on flow cytometry (600 Mb versus 750 Mb; [49]). The analysis of the Chinese pear genome [18] indicated that there may be significantly more repeated elements in the apple genome than in Chinese pear and our results in European pear validate this hypothesis.

### Comparative genomics between European pear and other plant species

A comparison of the predicted proteins in European pear was performed against the predicted proteins from 12 other plant species, including two *Rosaceae* pome fruit species: Chinese pear and apple. A caveat to interpretation of these results is that their precision depends both on that of the published proteomes and that of the predicted proteome of *P. communis*, wherein a potential bias could be introduced into the comparative analysis as a result of the 13 plant genomes being assembled and annotated by differing methodologies, as reported by the respective authors.

In European pear, we identified a subset of 556 clusters containing 1,219 proteins that did not have orthologs detected in the other 12 species used in the analysis. Further analysis of these proteins using a wider array of species for comparison would be required to determine whether these proteins encode for traits specific to European pear. Furthermore, the set of 1,433 protein clusters present in both pear species (1,684 and 1,905 proteins in European and Chinese pear, respectively) and apple (1,963 proteins) but not detected in the remainder of the species may include products of genes determining the pome fruit character. Further investigation, including RNA-seq analysis of developing fruit should be performed, to elucidate the genetic control of development of this unique fruit type.

### A tool for functional characterisation of fruit quality in pome fruit

The variation in fruit texture in pears is considerable, ranging from crisp in Chinese (*P. bretschneideri*) and Japanese (*P. pyrifolia*) pears, to melting in European pears. This melting texture does not occur in other pome fruit, such as apple and quince, which makes the study of comparative genomics of cell wall-related genes within the *Pyreae* very important. The role of expansins in fruit ripening was first demonstrated in tomato, where suppression and over-expression of ripening-specific *LeEXP1* was shown to result in increased fruit firmness and enhanced fruit softening, respectively [52]. In apple and pear, the involvement of expansins in the determination of fruit texture has also been inferred from expression analysis of ripening-related members that correlate with changes in fruit firmness [53], [54]. Our analysis of the expansin-like gene family indicated that the European pear and apple expansin gene families are of similar size (41 and 49 genes, respectively), which suggests that clade expansion has not occurred within either species. Only a few α-expansins (EXPA clade) appear to be associated with fruit softening, with one clade containing *PcEXP1*,*2* and *3* exhibiting high expression (Figure 3A) The expression analysis presented here confirms previous studies where *PcEXP1* to *PcEXP6*, but not *PcEXP7*, were highly expressed in cold-stored, ripening European pear [53], [55], and where *MdEXP3* was found to be the predominant, ripening-related expansin gene in apple [54], [56], [57]. Surprisingly, quantitative trait locus analysis linked *MdEXP7* to fruit softening in apple and pear [58], although *MdEXP7* expression was subsequently found to be undetectably low in a range of ripening apple genotypes [57]. Similarly in European pear, both in the current study and in [53], *PcEXP7* was one of the members of the family with very low expression (Figure 2A). Further examination of differences among the cultivars chosen for these different studies is required to further elucidate the role of expansins in fruit ripening in the *Pyreae*.

### The draft genome assembly of ‘Bartlett’ will contribute to faster delivery of new Pyrus cultivars

In the immediate future, the Bartlett v1.0 draft genome can be used as a reference for re-sequencing in *Pyrus* germplasm, as has been performed for apple [31] and peach [59]. Such germplasm re-sequencing will enable the development of high-throughput genetic marker screening tools for pear breeders, including SNP arrays and will also allow implementation of emerging technologies, such as genotyping by sequencing [60]. Such technologies will in turn enable the implementation of association studies for determination of marker-trait associations, as well as genomic selection (GS). Recent evaluation of genomic selection for fruit quality traits in apple indicates that genetic gains achievable using GS for a combination of traits, will be faster and more efficient than achieved by classical breeding [33], [61]. We predict that the availability of the ‘Bartlett’ draft genome sequence will enable the implementation of GS in pear cultivar breeding programmes internationally in the very near future.

## Supporting Information

Figure S1 Strategy used for anchoring the Bartlett v1.0 genome sequence.
(PPTX)

Table S1 Raw 454 sequencing data used to construct the Bartlett v1.0 genome sequence.
(XLSX)

Table S2 Analysis of the Core Eukaryotic Genes (CEGs; [30]) in the Bartlett v1.0 genome sequence.
(XLSX)

Table S3 Number of ortholog groups and genes in 13 plant species.
(XLSX)

Table S4 Anchoring of the Bartlett v1.0 genome sequence scaffolds on genetic maps constructed for apple and pear. Segregating populations used for genetic map construction: *Pyrus communis* family: ‘Old Home’×‘Louise de Bonne Jersey’; inter-specific Asian×European pear populations: NZSelection_pearT003(b)×‘Moonglow’, NZSelection_pearT042×NZSelection_pearT081 and NZSelection_pearT052×NZSelection_pearT003(a); apple segregating populations: PremA153×NZSelection_appleT031, ‘Fuji’×NZSelection_appleT051 and ‘Sciros’×NZSelection_appleT051 [33]. LG: Linkage Group.
(XLSX)

Table S5 List of gene models unique to European pear and their putative function.
(XLSX)

Table S6 Gene names and GenBank accession numbers for expansin gene models in European pear. LG: Linkage Group.
(DOCX)
