# Supplementary material for: The Draft Genome Sequence of European Pear (Pyrus communis L. ‘Bartlett’)
Source: PLoS One. 2014 Apr 3;9(4):e92644. doi: 10.1371/journal.pone.0092644 (PMC3974708; doi:10.1371/journal.pone.0092644)
Supplement: Table S6 — Gene names and GenBank accession numbers for expansin gene models in European pear. LG: Linkage Group. (DOCX) [file pone.0092644.s007.docx]

**Supplemental material 5** Gene names and GenBank accession numbers for expansin gene models in pear.

| **Expansin Type** | **Gene model name** | **Linkage group** | **Gene Name** | **GenBank Reference(s)** |
| --- | --- | --- | --- | --- |
| alpha | AUG2gene00033441 | LG7 | PcEXP1 | AB093028.1 |
| alpha | AUG2gene00015767 | LG1 | PcEXP2 | AB093029.1 |
| alpha | AUG2gene00003798 | LG-NA | PcEXP3 | AB093030.1; AJ811692.1* |
| alpha | AUG2gene00018787 | LG16 | PcEXP4 | AB093031.1 |
| alpha | AUG2gene00005316 | LG13 | PcEXP5 | AB093032.1 |
| alpha | AUG2gene00049304 | LG-NA | PcEXP6 | AB093033.1; AJ811691.1* |
| alpha | AUG2gene00034002 | LG-NA | PcEXP7 | AB093034.1 |
| alpha | AUG2gene00000192 | LG9 | PcEXPA8 |  |
| alpha | AUG2gene00010483 | LG14 | PcEXPA9 |  |
| alpha | AUG2gene00027134 | LG-NA | PcEXPA10 |  |
| alpha | AUG2gene00020125 | LG-NA | PcEXPA11 |  |
| alpha | AUG2gene00041836 | LG17 | PcEXPA12 |  |
| alpha | AUG2gene00039739 | LG-NA | PcEXPA13 |  |
| alpha | AUG2gene00014423 | LG-NA | PcEXPA14 |  |
| alpha | AUG2gene00036519 | LG11 | PcEXPA15 |  |
| alpha | AUG2gene00010398 | LG4 | PcEXPA17 |  |
| alpha | AUG2gene00030919 | LG8 | PcEXPA18 |  |
| alpha | AUG2gene00047550 | LG-NA | PcEXPA19 |  |
| alpha | AUG2gene00026567 | LG-NA | PcEXPA20 |  |
| alpha | AUG2gene00003350 | LG-NA | PcEXPA103 |  |
| alpha | AUG2gene00013190 | LG11 | PcEXPA104 |  |
| alpha | AUG2gene00005373 | LG12 | PcEXPA106 |  |
| alpha | AUG2gene00041466 | LG1 | PcEXPA107 |  |
| alpha | AUG2gene00041950 | LG-NA | PcEXPA108 |  |
| alpha | AUG2gene00008100 | LG6 | PcEXPA109 |  |
| alpha | AUG2gene00008571 | LG10 | PcEXPA110 |  |
| alpha | AUG2gene00032198 | LG-NA | PcEXPA111 |  |
| alpha | AUG2gene00008734 | LG2 | PcEXPA112 |  |
| alpha | AUG2gene00013452 | LG12 | PcEXPA113 |  |
| alpha | AUG2gene00049003 | LG3 | PcEXPA115 |  |
| alpha | AUG2gene00040828 | LG15 | PcEXPA118 |  |
| alpha | AUG2gene00017174 | LG-NA | PcEXPA119 |  |
| alpha | AUG2gene00009340 | LG-NA | PcEXPA213 |  |
| beta | AUG2gene00011051 | LG-NA | PcEXPB1 |  |
| beta | AUG2gene00035968 | LG7 | PcEXPB2 |  |
| beta | AUG2gene00002987 | LG1 | PcEXPB102 |  |
| alpha-like | AUG2gene00024475 | LG-NA | PcEXLA1 |  |
| alpha-like | AUG2gene00016830 | LG-NA | PcEXLA101 |  |
| beta-like | AUG2gene00046498 | LG-NA | PcEXLB1 |  |
| beta-like | AUG2gene00003119 | LG-NA | PcEXLB101 |  |
| beta-like | AUG2gene00026237 | LG6 | PcEXLB2 |  |
| * Gene names of AB-prefixed GenBank references (submitted 2002) used as AJ-prefixed references submitted later in 2004. | | | | |

**Supplemental material 5** Gene names and GenBank accession numbers for expansin gene models in apple.

| **Expansin Type** | **Gene model name** | **Linkage group** | **Gene Name** | **GenBank Reference(s)** | **Alternate gene model(s) at loci** |
| --- | --- | --- | --- | --- | --- |
| alpha | MDP0000138500 | LG11 | MdEXPA1 | AB099928.1; AY083166.1 |  |
| alpha | MDP0000431696 | LG1 | MdEXPA2 | AB099927.1; AY083167.1 | MDP0000772420 |
| alpha | MDP0000670959 | LG17 | MdEXPA3 | AB099926.1; AF527800.1 |  |
| alpha | MDP0000681724 | LG16 | MdEXPA4 | AB099925.1 |  |
| alpha | MDP0000432497 | LG9 | MdEXPA5 | AB099929.1 | MDP0000574554 |
| alpha | MDP0000195798 | LG1 | MdEXPA6 | AB099930.1 |  |
| alpha | MDP0000573617 | LG1 | MdEXPA7 | DQ072009.1 |  |
| alpha | MDP0000521662 | LG4 | MdEXPA8 | HQ905438.1 |  |
| alpha | MDP0000193127 | LG6 | MdEXPA9 |  |  |
| alpha | MDP0000496071 | LG8 | MdEXPA10 |  | MDP0000249929 |
| alpha | MDP0000123429 | LG17 | MdEXPA11 |  | MDP0000132870 |
| alpha | MDP0000261103 | LG-NA | MdEXPA12 |  |  |
| alpha | MDP0000238208 | LG12 | MdEXPA13 |  | MDP0000931842 |
| alpha | MDP0000123864 | LG12 | MdEXPA14 |  |  |
| alpha | MDP0000504183 | LG11 | MdEXPA15 |  |  |
| alpha | MDP0000410264 | LG4 | MdEXPA16 |  | MDP0000817926 |
| alpha | MDP0000765613 | LG12 | MdEXPA17 |  |  |
| alpha | MDP0000240613 | LG8 | MdEXPA18 |  | MDP0000134342 |
| alpha | MDP0000443876 | LG3 | MdEXPA19 |  |  |
| alpha | MDP0000273450 | LG11 | MdEXPA20 |  |  |
| alpha | MDP0000155768 | LG2 | MdEXPA21 |  |  |
| alpha | MDP0000228494 | LG3 | MdEXPA101 |  |  |
| alpha | MDP0000560112 | LG13 | MdEXPA104 |  |  |
| alpha | MDP0000151618 | LG14 | MdEXPA109 |  | MDP0000193025 |
| alpha | MDP0000192586 | LG10 | MdEXPA110 |  |  |
| alpha | MDP0000259640 | LG9 | MdEXPA111 |  | MDP0000785413 |
| alpha | MDP0000679320 | LG9 | MdEXPA112 |  |  |
| alpha | MDP0000157370 | LG12 | MdEXPA113 |  |  |
| alpha | MDP0000715154 | LG12 | MdEXPA114 |  |  |
| alpha | MDP0000697206 | LG3 | MdEXPA115 |  |  |
| alpha | MDP0000743239 | LG12 | MdEXPA116 |  |  |
| alpha | MDP0000858585 | LG4 | MdEXPA117 |  | MDP0000627765 |
| alpha | MDP0000615769 | LG12 | MdEXPA118 |  |  |
| alpha | MDP0000258359 | LG10 | MdEXPA119 |  | MDP0000686217 |
| alpha | MDP0000219482 | LG-NA | MdEXPA120 |  |  |
| alpha | MDP0000156815 | LG12 | MdEXPA121 |  | MDP0000812586 |
| alpha | MDP0000420893 | LG2 | MdEXPA212 |  | MDP0000620349 |
| alpha | MDP0000192434 | LG12 | MdEXPA213 |  |  |
| alpha | MDP0000257797 | LG1 | MdEXPA215 |  |  |
| alpha | MDP0000306058 | LG3 | MdEXPA220 |  | MDP0000128827 |
| beta | MDP0000435467 | LG17 | MdEXPB1 |  |  |
| beta | MDP0000695604 | LG-NA | MdEXPB2 |  |  |
| beta | MDP0000437664 | LG8 | MdEXPB101 |  | MDP0000147368 |
| beta | MDP0000290170 | LG7 | MdEXPB102 |  |  |
| alpha-like | MDP0000126245 | LG9 | MdEXLA101 |  |  |
| alpha-like | MDP0000568045 | LG10 | MdEXLA1 |  |  |
| beta-like | MDP0000165364 | LG6 | MdEXLB1 |  | MDP0000640549 |
| beta-like | MDP0000214811 | LG6 | MdEXLB2 |  | MDP0000292477; MDP0000906812 |
| beta-like | MDP0000294444 | LG16 | MdEXLB101 |  |  |
